# Determining the coverage and efficacy of the COVID-19 vaccination program at the community level in children aged 12 to 17 in Tehran

**DOI:** 10.3205/dgkh000459

**Published:** 2024-01-30

**Authors:** Sedigheh Rafiei Tabatabaei, Delara Babaie, Seyedeh Mahsan Hoseini-Alfatemi, Ahmadreza Shamshiri, Abdollah Karimi

**Affiliations:** 1 Pediatric Infections Research Center, Research Institute for Children’s Health, Shahid Beheshti University of Medical Sciences, Tehran, Iran; 2 Department of Allergy and Clinical Immunology, Mofid Children’s Hospital, Shahid Beheshti University of Medical Sciences, Tehran, Iran; 3 Research Center for Caries Prevention, Dentistry Research Institute, Department of Community Oral Health, School of Dentistry, Tehran University of Medical Sciences, Tehran, Iran

**Keywords:** COVID 19, vaccination coverage, child, hospitalized

## Abstract

**Background:**

The vaccination is one of the acceptable and recomended solution to prevent and control of COVID-19. The aim of this study was to determine the efficacy of sinopharm vaccination in children aged 12–17 in Tehran.

**Methods:**

The case population study was performed from October 2021 to March 2022 among 1,500 children with positive PCR test reffered in Mofid Children’s Hospital in Tehran. 64 children aged 12–17 years were included. The data were collected by the hospital information system (HIS), vaccination information registration systems and questionnaire with their families. The coverage and efficacy of vaccination determined with equels commented by WHO.

**Results:**

Out of 64 children, 52 children were 12 to 15 years old (13.35±1.08), 12 children were 16 to 17 years old (16.55±0.52). 48.4% had received two doses of vaccine. The highest rate of positive PCR was observed in February 2022. Sinopharm vaccine coverage in this age group was 93.6% for the first dose and 81.1% for the second dose. Based on this information, 48.4% children in this study have received two complete doses of the COVID-19 vaccine. The efficacy of the vaccine was estimated as 94.4% (95% CI 90.2 to 97.7).

**Conclusion:**

It seems the coverage of Sinopharm vaccination in the age group of 12–17 years in Tehran is favorable and has high efficacy in this age group. In order to obtain more accurate and comprehensive estimation, it is recommended to take a sample on a wider level of the community.

## Zusammenfassung

**Hintergrund:**

Die Schutzimpfung ist eine der akzeptablen und empfohlenen Lösungen zur Prävention und Kontrolle von COVID-19. Ziel dieser Studie war es, die Wirksamkeit der Sinopharm-Impfung bei Kindern im Alter von 12-17 Jahren in Teheran zu ermitteln.

**Methode:**

Die Fallpopulationsstudie wurde von Oktober 2021 bis März 2022 im Mofid-Kinderkrankenhaus in Teheran unter 1.500 Kindern mit positivem PCR Test durchgeführt. Es wurden 64 Kinder im Alter von 12–17 Jahren, die mit Sinopharm geimpft wurden, in die Studie eingeschlossen. Die Daten wurden mit Hilfe des Krankenhausinformationssystems (KIS), des Registrierungssystems für Impfungen und eines Fragebogens für die Familien erhoben. Die Durchimpfungsrate und die Wirksamkeit der Impfung wurden anhand der von der WHO kommentierten Gleichungen ermittelt.

**Ergebnisse:**

Von den 64 Kindern waren 52 Kinder 12 bis 15 Jahre alt (13,4±1,08); 12 Kinder waren 16 bis 17 Jahre alt (16,6±0,52). 48,4% hatten zwei Dosen des Impfstoffs erhalten. Die höchste Rate an positiven PCRs wurde im Februar 2022 beobachtet. Die Durchimpfungsrate mit Sinopharm betrug in dieser Altersgruppe 93,6% für die erste Dosis und 81,1% für die zweite Dosis. Auf der Grundlage dieser Informationen haben 48,4% der Kinder in dieser Studie zwei vollständige Dosen des COVID-19-Impfstoffs erhalten; die Wirksamkeit des Impfstoffs wurde auf 94,4% (95% CI 90,2 bis 97,7) geschätzt.

**Schlussfolgerung:**

Es scheint, dass die Durchimpfungsrate der Sinopharm-Impfung in der Altersgruppe der 12- bis 17-Jährigen in Teheran günstig ist und die Impfung eine hohe Wirksamkeit in dieser Altersgruppe aufweist. Um eine genauere und umfassendere Schätzung zu erhalten, wird empfohlen, eine Stichprobe auf einer breiteren Ebene der Gemeinschaft zu nehmen.

## Introduction

With the spread of COVID-19, transmission reduction strategies such as physical distancing were used in parallel with the use of drug therapy [1], [2], [3] to treat the disease. Although this strategy was effective in reducing infection rates, it did not protect individuals, especially vulnerable groups, from infection. Therefore, the best way to control this epidemic infection was to create a useful and effective vaccine [4], [5]. The vaccine is a solution used in the past to reduce mortality from infectious diseases and can be effective as an option to stop this pandemic. Currently, more than 100 COVID-19 vaccines are in development, and 11 vaccines have been placed on the Emergency Use List (EUL) by World Health Organization (WHO) [6].

In general, four types of vaccines have been considered in the COVID-19 pandemic. Inactivated vaccines (Sinopharm), nucleic acid-based vaccines or messenger ribonucleic acid (mRNA) vaccines (Pfizer and Moderna), vector-based vaccines and subunit vaccines [4], [7], [8], [9]. Each vaccine has different storage conditions, strengths and duration of efficacy depending on the antigen design, adjuvant molecules, vaccine delivery platforms, and immunization method. Until January 2023, 12 types of vaccines have been used in the country vaccination [10].

According to the WHO data, until January 30, 2023, a total of 13,168,935,724 doses of vaccine (69.4% of the population) has been injected globally, with 26% of people in low-income countries at least one dosage [11], [12]. These data are obtained based on the reports announced by governments and ministries of health around the world. In Iran, until January 28, 2023, a total of 155,011,109 vaccine doses [12], [13], [14] was injected. 

Although the severe prevalence of COVID-19 and hospitalization is higher among adults, according to various studies, these consequences have also been reported in the children and adolescents groups [15], [16], [17], [18], especially in those with underling health conditions [19], [20]. On the other hand, one study found a similar prevalence between children and adults. This study was shown a higher proportion of infections in children appears to be asymptomatic [21]. Also, one study was shown, about one-third of 12- to 17-year-olds hospitalized for COVID-19 from March 2020 to April 2021 in United States required intensive care unit (ICU) and 5% required endotracheal intubation and mechanical ventilation [18]. In addition, the psycho-social consequences of COVID-19 on children and adolescents are a critical reason to control this epidemic more effectively under 18 years of age [22]. School closures reduced interaction with peers, opportunities for physical activity, and children’s learning progress at home. In this way, the social functioning of about 1.5 billion young people worldwide was affected by this disease [23]. Therefore, the Food and Drug Administration (FDA) issued an Emergency Use Authorization (EUA) for the Pfizer-BioNTech COVID-19 vaccine for adolescents aged 16 to 17 years on December 11, 2020, in the United States. The EUA was extended to 12–15 year olds on 10 May, 2021 [15], following the emergence of the delta variant and the increase in pediatric hospitalizations by CDC’s Advisory Committee on Immunization Practices [24].

Among US adolescents aged 12–17 years, as of July 31, 2021, COVID-19 vaccination coverage was 42.4% for ≥1 dose and 31.9% for completing the series [15]. Series completion is defined as receiving both doses of the Pfizer-BioNTech or Moderna vaccine, and those who with mismatched products receiving between the first and second doses or a single dose of Janssen vaccine [15].

By understanding and describing the epidemiology of hospitalizations associated with COVID-19 and comparing it to other vaccine-preventable respiratory viruses, the broad age-recommended benefits of vaccination are presented and also, the impact of vaccination is evaluated [18]. As a result, using the 2019 Corona Disease-Related Hospitalization Surveillance Network (COVID-NET) – a population-based surveillance system of laboratory-confirmed COVID-19-associated hospitalizations in 99 counties across 14 state in US – CDC analyzed the hospitalizations associated with COVID-19 among 12 to 17 years, including demographic and clinical characteristics of adolescents hospitalized from January 1 to March 31, 2021, and also examined hospitalization rates (hospitalizations per 100,000 population) among adolescents from March 1, 2020, to April 24, 2021 [18]. During March 1, 2020, through April 24, 2021, the weekly adolescent hospitalization rate peaked at 2.1 per 100,000 in early January 2021, decreased to 0.6 in mid-March, and then increased to 1.3 in April [18].

Despite the many studies conducted about the coverage and efficacy of vaccinations under 18 years old [15], [16], [23], [24], [25] in the world, there is little information about the efficacy and coverage of vaccination in the age group especially under 18 years old in Iran. The statistics show the status of vaccination in the population, but there is no data based on age groups and the type of vaccine used in Iran [13]. Therefore, a preventive approach to control this disease in them should be considered. This study was conducted to estimate coverage and efficacy of the SARS-CoV-2 vaccination in children aged 12 to 17 living in Tehran.

## Method

### Study location

The study was conducted in Mofid Children’s Hospital in Tehran, Iran. 

### Study design

The case population study was performed as cross-sectional study from October 7, 2021, to March 20, 2022, among 1,500 children with positive PCR test reffered in Mofid Children’s Hospital in Tehran. The sampling was done by non-random consecutive sampling method. No formula was used to estimate the sample size. Based on haphazard sampling, all eligible people with the inclusion criteria were accepted into the study.

### Study population

The inclusion criteria consisted of children aged 12 to 17 who lived in Tehran and visited Mofid Children’s Hospital since the start of the COVID-19 vaccination in October 7, 2021, and their PCR test was positive. Children who did not live in Tehran or did not agree to participate in the study were excluded from the study. 

### Outcome variables

Two main outcome variables were evaluated. The coverage of vaccination in first and second dose in 2021 and 2022 in two age groups (12–15 and 16–17 y), and the efficacy of vaccination in 12 to 17 years old in Tehran. Also, some side outcomes were evaluated in participants, including; Type of vaccine, underlying disease and some laboratory signs and symptoms in hospitalized children in Mofid’s hospital in Tehran. 

### Data collection

Among 1,500 children with positive PCR tests referring to the hospital, 64 children were included in the study. The individual data including age, gender, underlying disease, place of residence, history of COVID-19 disease, laboratory parameters and disease symptoms, and type and time of vaccination were collected from the HIS, vaccination information registration systems and patient diagnostic information registration (laboratory and inpatient center), and also a telephone questionnaire from children’s families. The aggregate data including vaccination coverage and efficacy information were collected from the network management center – taken from the information of Iran Statistics Center in 2021. The participants were divided into two groups of 12–15 and 16–17 years old, and in terms of vaccination in two groups vaccinated at least 0 to 1 dose and at least 2 and more doses. The term “vaccinated” refers to people who have been vaccinated for at least 15 days. Therefore, 0 to 1 dose refers to children who visited the hospital less than 15 days after vaccination.

### Statistical analysis

The data were entered and analyzed in IBM SPSS 25. Quantitative variables were reported as mean ± standard deviation (SD) and qualitative variables as percentage and frequency. Vaccine coverage was calculated by dividing the number of children 12 to 17 years old vaccinated by the population of children 12 to 17 year’s old living in Tehran province. 

### Ethical approval

The study was conducted per the declarations of Helsinki. Ethical approval was obtained from the ethical committee of the Research Institute for Children’s Health, Shahid Beheshti University of medical science (IR.SBMU.RICH.REC.1400.058). Ethics committee approved permission for verbal consent, then informed consent was obtained from their legal guardian (verbal based on local ethics requirements).

### Equations

The WHO efficacy equations of vaccination [26] were used to determine the vaccination efficacy. These equations are shown in Equation 1.


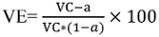


**Equation 1: Vaccine efficacy; VE=vaccine efficacy, VC=vaccine coverage%; a=percentage of vaccinated cases**

## Results

### Basic characteristics of participants

Of the 64 children aged 12 to 17 who entered the study, 72.7% (n=64, 29 girls and 35 boys) were living of Tehran. There were 52 children in 12–15 years group and 12 children in 16–17 years group. The underlying disease was reported in 39.1% of children (n=25). Diabetes (n=4), nephrotic syndrome (n=2) and cerebral palsy (n=2) are highest.

According to the parent’s reports, 53.1% of children (n=34) received the first and 48.4% of them (n=31) recieved the second doses of Sinopharm vaccine. Also, in each first and second dosages, 1 child was vaccinated with AstraZeneca vaccine and one child was vaccinated with Pastcovac (Soberana) vaccine. According to the available data, 28 children had not received the vaccine and one case had an unknown vaccination status. Also, of the participants who had an underlying disease, 9 people were vaccinated with at least one dose and 5 people with two full doses. The basic characteristics of children are presented by two age groups in Table 1 (Tab. 1).

Also, the children with positive PCR tests by month incorporated 28% in October, 14% in November, 3% in December, 1.5% in January, 30% in February, 12.5% in March, 3% in April, and 8% in May. Based on these findings, the highest rate of positive PCR was observed in February 2022 and then in October 2021.

### Symptoms and laboratory tests data 

Of all 64 children with positive PCR test, disease symptoms were recorded and laboratory tests were performed. Most of the symptoms of the disease were observed in the 12–15 years group, which mostly included fever (n=19, 29.7%), cough (n=12, 18.8%) and vomiting (n=6, 9.4%). In the age group of 16–17 years, vomiting (n=2), headache, shortness of breath (n=1) and nausea (n=1) were reported. In laboratory test, the total mean ± SD of white blood count (WBC), C-reactive protein (CRP), erythrocyte sedimentation rate (ESR) and aspartate aminotransferase (AST) in two groups was 8,820.00±6,975.50, 18.42±14.70, 27.12±20.44, and 43.60±44.71, respectively. 

### Vaccination coverage 

**Figure 1:**
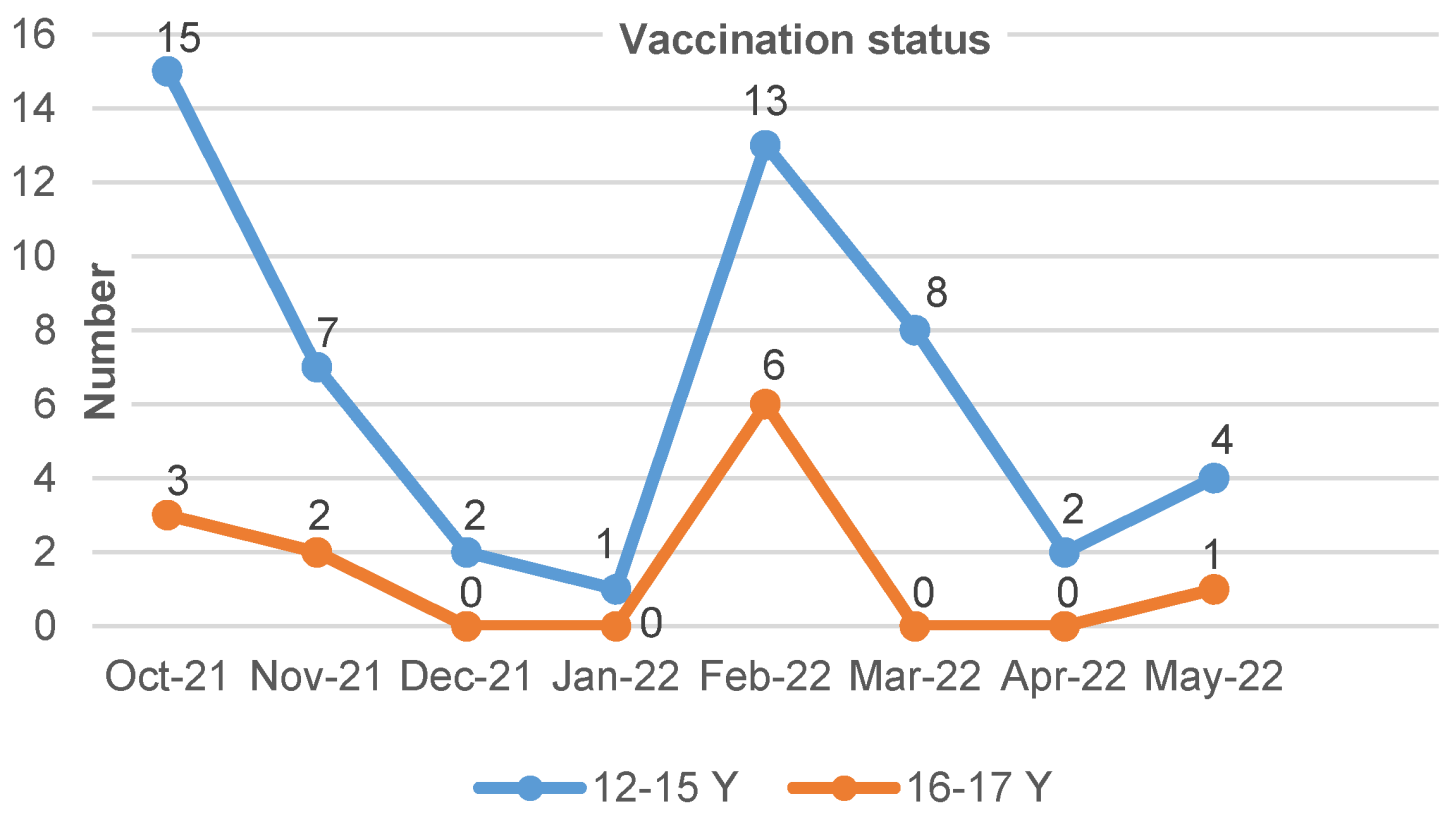
Vaccination status by months and age groups in patient with positive PCR admitted in Mofid hospital

In order to determine the vaccination coverage, the number of vaccinated children at the end of each study month (October 2021 to March 2022) by the age groups of 12–15 and 16–17 years old was extracted based on hospital data. These data are presented as children’s vaccination status by month and age groups in Table 2 (Tab. 2), Table 3 (Tab. 3) and Figure 1 (Fig. 1).

As shown in Table 2 (Tab. 2) and Figure 1 (Fig. 1), the number of vaccinations in the age group of 12–15 years is more than the age group of 16–17 years, and in the months of December, January, March and April, none of the 16–17 years group had received vaccines.

In Table 3 (Tab. 3), the status of vaccination coverage for the age group of 12 to 17 years is presented. According to this table, the coverage percentage is 93.68% in the first and 81.1% in the second dose. This information has been extracted based on the documents available in the COVID-19 vaccination system in Tehran province. The population living in Tehran province is covered by the three universities of Tehran, Iran and Shahid Beheshti, which were included in these statistics.

### Vaccination efficacy

In Table 4 (Tab. 4), the vaccination status is presented by age group and dose, in nine months. Based on this table, a total of 64 children were vaccinated. The children aged 12–15 Y (n=52) were vaccinated more than children aged 16–17 Y (n=12). The vaccination rate in May 2022 was more than the December 2021 and Februray 2022. Also, the number of children who received the first dose of vaccine (n=50) is more than the number of children who received two or more doses of vaccine (n=14).

The efficacy of vaccination in the age group of 12–17 years in Tehran province based on the equations used is presented in Table 5 (Tab. 5). According to the findings, the efficacy of Sinopharm vaccine in children aged 12–17 years who have received at least two doses of the vaccine is equal to 94.4% (95% CI; 90.2%–97.7%). 

## Discussion

This study investigated the coverage and efficacy of vaccination in children aged 12–17 living in Tehran. The results of this study showed 93.6% and 81.1% of children aged 12–17 living in Tehran province were vaccinated against COVID-19 in the first and second dose, respectively. Also, this study showed the Sinopharm VE is 94.4% in vaccinated by at least two doses.

According to the statement of the Independent Vaccine Allocation Group (IAVG) COVAX, the achievement of 70% immunization coverage for COVID-19 was identified as a necessity by mid-2022 [27]. This coverage target was set to ensure an equitable pace of global vaccine supply and to prioritize those at the highest risk [27]. Based on NYT data in October 2022, the percentage of fully-vaccinated residents in 12–17 years in the U.S. is 61% in total with the least in Wyoming (36%) and the most in Puerto Rico (93%) [28]. Also, all 30 countries of the European Union and the European Economic Area (EU/EEA) recommend primary vaccination against COVID-19 for adolescents aged 12–17 years. According to countries reports, in EU/EEA countries, as of 30 January 2022, the median primary dose of the COVID-19 vaccine was 70.9% among those aged 15–17 years based in 17 countries and 35.5% among 10–14 years in 16 countries. More than half of adolescents aged 10–17 have not yet completed a primary dose in the EU/EEA [29]. According to these reports, progress in vaccine uptake is unequal in EU/EEA countries [30]. 

In Iran, vaccination of under 18 years started in the fall of 2021. According to our findings, the vaccination rate in the participants in the first month of vaccination (October 2021) was higher in this age group (n=18) and then had a decreasing trend. In February 2022 – with the outbreak of Omicron in Iran – the number of vaccinated participants increased (n=19), which was more in the 12–15 years group compared to the 16–17 years group. This can be due to the higher number of participants in this age group compared to the age group of 16–17 years. Generally, 53.5% of vaccination was from February to May because Omicron started at the end of January.

According to previous studies, as vaccination coverage rates increase, especially if the coverage rate is greater than 60%, the number of new cases per million population, new deaths per million population, and hospital or ICU patients per million population, as well as the reproduction rate of COVID-19, will be gradually decreased, and benefits of preventing severe illness and preventing transmission of infection will become apparent [31]. Although, despite this importance, vaccination trends are progressing slowly around the world [32]. According to the global distribution statistics of the epidemiology and vaccination rate of COVID-19 until 20 August, 2021, the average number of vaccinated people per 100 was 40.8%, and European countries with 54.6% were the highest, followed by North America (48.2%), Asia (45.7%), South America (45.2%), Oceania (42.5%) and Africa (20.5%) were respectively in the next positions of vaccination of COVID-19. In this way, it seems this low coverage rate is too low to effectively prevent the spread of the epidemic [33] that commented with COVAX [27]. Other studies reported vaccination coverage of 81% in the Lazio region of Italy [34] and 67% in Hong Kong [35], with coverage rates decreasing with age. In Iran, 79% of people are vaccinated with at least one dose, and 71% are fully vaccinated. Although these values are related to vaccination in all age groups, it shows that the world is still far from the determined vaccination coverage value.

In this way, comparing the statistics with these findings shows vaccination coverage is higher in Iran. However, since this study was conducted with a smaller sampling and in one province, there is a possibility of bias due to the differential exposure of vaccinated and unvaccinated individuals. Therefore, the results of this study cannot be generalized to the Tehran province or the whole country. 

The vaccine efficacy indicates the percentage reduction in the risk or odds of disease or infection in vaccinated individuals [36]. To evaluate the efficacy of COVID-19 vaccines in different populations and environments, it is necessary to measure the performance of these vaccines in preventing symptomatic disease, severe disease, infection and transmission, hospitalization, death, and other outcomes [36]. Evidence showed that primary vaccination against the delta variant increased protection against infection, symptomatic disease, and severe disease in adolescents. This evidence has also revealed that the vaccine efficacy against symptomatic infections declines 5 to 6 months after completion of the primary vaccination course. Primary vaccination seems to be highly effective against severe outcomes including hospitalization due to the delta variant. But there is no evidence about the length of protection [29]. In the current study, the efficacy of Sinopharm vaccination was 94.4% following at least two doses in the age group of 17–12 years. 

Large-scale clinical studies show that although the COVID-19 vaccines prevent most people from getting COVID-19, they are not 100% effective like most other vaccines [37]. Based on these studies, this vaccine has relatively low antibody levels but is well tolerated [37] and 100% effective in preventing mild and severe cases of COVID-19 [38]. But the severity of COVID-19 does not have a difference significantly following the first or second dose in infected patients [39]. In a case-control study of a negative test in England, which examined the vaccine efficacy in adolescents, it was shown that after 14 days of the first dose of Comirnaty in adolescents aged 16–17 years, the vaccine efficacy was 75.9%, gradually decreased, and after 8–9 weeks it reached 37.4% [40]. Also, a peer-controlled case study in the U.S. showed that the vaccine efficacy after two doses of Comirnaty vaccination could support severe outcomes such as hospitalization (94%), ICU admission (98%), and life-support intervention or death (98%) in aged 12–17 [25]. The delta variant was dominant in the above two studies [25], [41].

However, studies of the vaccine efficacy in this age group are relatively few, and estimates may be biased by distinguishing between vaccinated and unvaccinated individuals. It is also possible that most of the estimations are based on studies conducted with small sample sizes. Also, vaccine efficacy estimation may be affected by factors such as target population, different vaccine schedules, or bias and confounding, leading to different results.

Based on the available evidence, the evaluation of WBC, CRP, ESR, and AST levels in children with COVID-19 is an critical predictor for hospitalization as well as determining the severity of the infection [42]. According to the study of Armin et al. A WBC of more than 15,000, and CRP of more than 20 may increase the hospitalization time to 15 days. Also, based on the available evidence, the presence of underlying disease and CRP level are valuable predictive factors for the progression of the disease from mild to severe in adults [43]. In the current study, 39% of children had the underlying disease and the amount of WBC and CRP was in the normal range, which according to the findings of Armin et al. [42], the duration of hospitalization is reduced to 5 days. 

One of the challenges of this study was the difficulty of accessing the information of the study participants. Most of the data was obtained from the participants through medical records, country vaccination information, and phone calls to their families. Considering the efficacy of each vaccine, whether an additional dose is needed or not, or whether new vaccines are required to deal with the disease? These are questions that can be answered by evaluating the vaccine’s efficacy.

The main limitations of this study are the small sample size and the lack of statistics for the two age groups studied to determine the vaccination coverage, separately. Due to the small sample size, the obtained results cannot be generalized to the vaccination of this age group in the country. Also, because most of the participants in this study were vaccinated by the Sinopharm, it was necessary to compare the results of this study with similar studies. However, since few studies have been done in the field of Sinopharm vaccination in under 18 years, the results of this study cannot be compared with Sinopharm vaccination studies in other age groups. In addition, few vaccines have been suggested for age groups below 18 years, which have not been used in Iran. On the other hand, the WHO has introduced two methods, a retrospective cohort and case-control study with a negative test design [44], to determine the efficacy of the vaccine, while this study used the case population study method, which may provide different results from the above two methods. However, in retrospective cohort studies, there is a possibility of bias due to age, history of infection, geographic location, and social and economic status.

## Conclusion

This study investigated the coverage and efficacy of Sinopharm vaccination in the age group of 12–17 years. The findings indicate that compared to some countries, the vaccine coverage rate in Iran is favorable and has high efficacy in this age group. Estimating vaccine efficacy can be a solution to answer important questions and concerns in public health in the field of COVID-19.

## Limitations of the study

The limitation is the small number of children vaccinated with the Covid-19 vaccine admitted to the Children’s Hospital. It is suggested that future studies be conducted on the types of vaccines injected to children and in a longer period of time.

## Notes

### Competing interests

The authors declare that they have no competing interests.

### Acknowledgments

This research was supported by Shahid Beheshti University of Medical Sciences, Tehran, Iran. We would like to thank our colleagues from “Pediatric Infections Research Center, Research Institute for Children’s Health, Shahid Beheshti University of Medical Sciences, Tehran, Iran”, who greatly helped the research by conducting and providing the results of PCR tests for children.

### Funding

The study is supported by Shahid Beheshti University of Medical Sciences, Tehran, Iran. The funder had no role in the conception, design or planning of the study and will not have a role during conduct of the study; data collection, management, analysis, interpretation of the data or decision to publish.

### Authors’ ORCIDs

- Hoseini-Alfatemi SM: 0000-0002-8293-934X
- Ahmadreza S: 0000-0002-7170-0106

## Figures and Tables

**Table 1:**
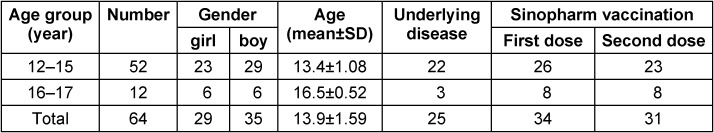
COVID patients with a positive PCR test admitted to the Mofid hospital from October 2021 to March 2022

**Table 2:**
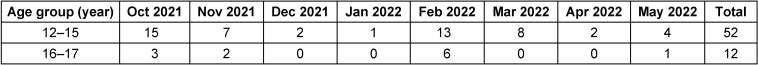
Vaccination status by months and age groups in COVID-19 patients with a positive PCR test admitted to the Mofid hospital from October 2021 to May 2022

**Table 3:**
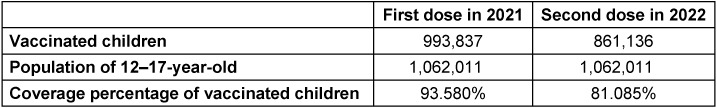
The coverage percentage of vaccined children (12–17 years) in Tehran province in 2021

**Table 4:**
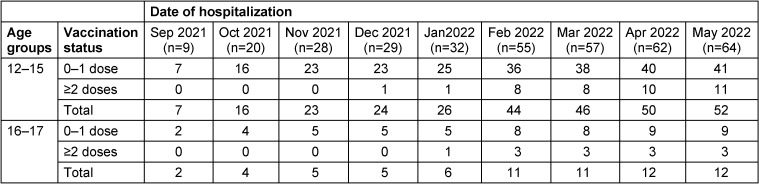
Vaccination status (at least 2 doses) in children by two age groups and time of hospitalization

**Table 5:**
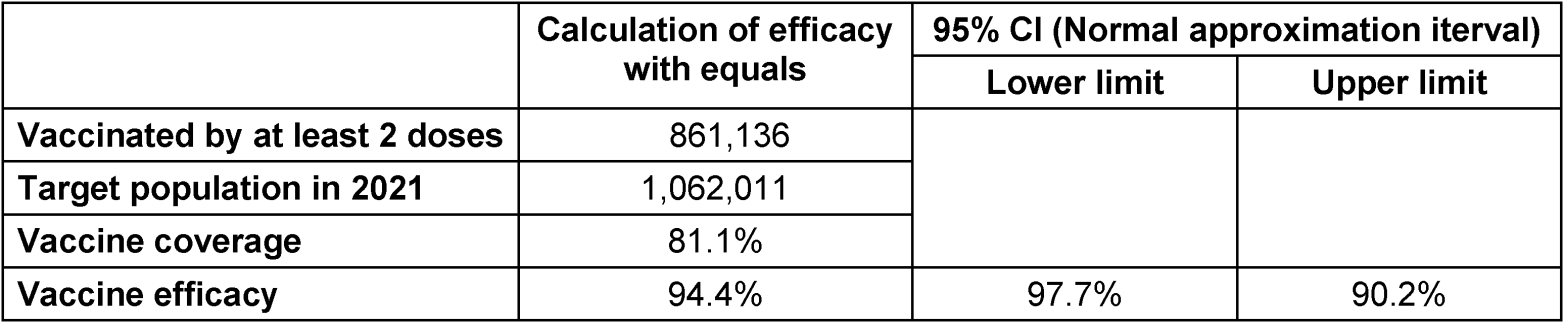
Efficacy of Sinopharm vaccine in 12–17 years in Tehran province
